# Comparison of Surface Persistence of SARS-CoV-2 Alpha and Delta Variants on Stainless Steel at 4°C and 24°C

**DOI:** 10.1128/aem.00764-22

**Published:** 2022-07-11

**Authors:** Okechukwu Onianwa, Isobel Garratt, Jennifer Carter, Antony Spencer, Neville Q. Verlander, Thomas Pottage, Allan M. Bennett

**Affiliations:** a Research and Evaluation, UK Health Security Agency, Porton Down, United Kingdom; b Statistics, Modelling and Economics Department, UK Health Security Agency, United Kingdom; University of Nebraska-Lincoln

**Keywords:** Alpha variant, COVID-19, Delta variant, fomites, RNA virus, SARS-CoV-2 variants, stainless steel, surface stability, surface persistence, winter transmission

## Abstract

Most studies on surface persistence of SARS-CoV-2 have been conducted at temperatures between 20°C and 30°C. There is limited data on the survival of SARS-CoV-2 at low temperatures. In this study, the stability of SARS-CoV-2 Alpha and Delta variants on stainless steel was investigated at two temperatures (4°C and 24°C). The results show that both variants decayed more rapidly at 24°C compared with 4°C. At 24°C, Alpha and Delta variants showed reductions of 0.33 log_10_ and 1.02 log_10_, respectively, within the first 2.5 h. However, at 4°C, Alpha variant showed a reduction of 0.16 log_10_ within the first 2.5 h while no reduction was observed with Delta variant. After remaining *in situ* for 24 h at 24°C, log_10_ reductions of 2.66 (Alpha) and 3.11 (Delta) were observed. No viable Alpha and Delta variant was recovered after 48 h and 72 h, respectively. After 24 h in a refrigerated environment (4°C) log_10_ reductions of 1.16 (Alpha) and 0.95 (Delta) were observed. Under these experimental conditions, both viruses survived on stainless steel for at least 1 week. No viable Alpha and Delta variant was recovered after 10 days. These findings support the potential for increased fomite transmission of SARS-CoV-2 during winter months in colder regions worldwide and in some industrial sectors.

**IMPORTANCE** Human transmission is believed to occur primarily through direct transfer of infectious droplets or aerosols. However, fomite transmission through contact with contaminated surfaces may also play an important role. This study provides novel evidence comparing the stability of Alpha and Delta variants on stainless steel surfaces at 4°C and 24°C. At 4°C both variants were found to be still detectable for up to 7 days. At 24°C Delta variant could be recovered over 2 days compared with Alpha variant which could not be recovered after 2 days. This has implications for fomite transmission interventions for people living and working in cold environments.

## INTRODUCTION

The first outbreak of SARS-CoV-2 was reported in Wuhan, China in the Autumn of 2019. A rapid increase in case numbers over the following months led to its declaration as a pandemic by the World Health Organization (1). Not long after, reports soon emerged of variants of concern which possess significant mutations (including N501Y and D614G), associated with increased transmissibility and disease severity, as well as reduction in antibody neutralization (2).

There is limited data on surface survival of SARS-CoV-2 at low temperatures (3, 4). Many studies on environmental persistence of SARS-CoV-2 on stainless steel have been carried out at temperatures ranging between 20°C and 30°C (4–9). While such investigations are relevant for countries with warmer climates, they do not apply to colder regions globally. Seasonal changes and temperature fluctuations may impact virus stability differently and to colder working environments such as food factories. To bridge this gap in knowledge, the surface stability of the Alpha and Delta SARS-CoV-2 variants of concern has been investigated under different experimental conditions.

## RESULTS AND DISCUSSION

Surface survival studies at cold temperatures are relevant for cold climates. They are also important in cold-chain transportation where contaminated food and associated packaging could present a risk for fomite transmission to workers (10, 11). Liu et al. recently screened frozen cod outer packaging for presence of SARS-CoV-2, following reports of asymptomatic infection among Stevedores. Viral RNA was detected in 12% of samples tested, and virus isolated from one sample (12).

In the present study, we report the persistence of SARS-CoV-2 Alpha and Delta variants on stainless steel at average conditions of relative humidity (RH) 4°C with >85% RH as well as 24°C with 63% RH. Stainless steel is a good representative carrier to use because of its inert properties. It also reflects the material used in the built environment, where, due to the high frequently of contact by individuals, is well-suited for surface stability studies of viruses, including SARS-CoV-2. It also provides an inanimate surface that SARS-CoV-2 has been reported to persist on (4, 5).

**FIG 1:**
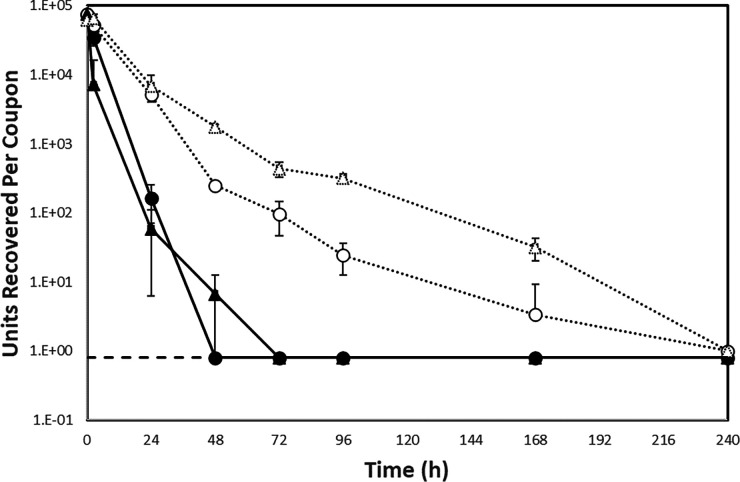
Mean titers of viable virus recovered from stainless-steel coupons inoculated with SARS-CoV-2 Alpha variant (circles) and Delta variant (triangles). Circles and triangles are represented as black or white based on temperature and humidity (black, 24°C and 63% RH; white, 4°C and >85%). Error bars represent standard deviation from three replicates. Dashed line represents limit of detection (0.8 pfu/mL).

At 4°C, Alpha variant showed a reduction of 0.16 log_10_ within the first 2.5 h while no reduction was observed with Delta variant. The samples were still visibly wet within the first 2.5 h at 4°C, but dry within 24 h. However, when dried at 24°C, a sharper drop in viability was noticed with both variants. Alpha variant showed a reduction of 0.33 log_10_ within the first 2.5 h, while Delta variant had a reduction of 1.02 log_10_ (Fig. 1). The rate of inactivation with both variants decreased after the initial drying period. This biphasic decay has also been reported by other groups (3, 4, 8, 9).

Although starting quantities for both variants were similar (Alpha variant at 6.2 × 10^4^ PFU and Delta variant at 1.56 × 10^4^ PFU), at 24°C, log_10_ reductions of 2.66 (Alpha) and 3.11 (Delta) were observed. No viable Alpha and Delta variant was recovered after 48 h and 72 h, respectively (Fig. 1), showing that SARS-CoV-2 Delta variant is more stable at room temperature under the conditions used in this study compared to the Alpha variant. After 24 h in a refrigerated environment (4°C) log_10_ reductions of 1.16 (Alpha) and 0.95 (Delta) were observed. Under these experimental conditions, both viruses survived on stainless steel for at least 1 week. No viable virus was recovered by day 10. Van Doremalen et al. conducted a similar study investigating persistence in closely related MERS-CoV isolates obtained from human and camels. C/KSA/13, an isolate with polymorphisms in ORF1b, the spike glycoprotein, and the virion matrix protein, exhibited reduced surface stability compared with other MERS-CoV strains tested (13). Investigations on the possible link between polymorphisms and surface persistence are required.

When excluding time point 0, a linear function was adequate. The trend in viability with each variant differed significantly according to the temperature (*p* <0.001 for three-way interaction), with a more marked decrease in viability at 24°C compared with 4°C. The difference was greater for Alpha compared with Delta. Thus, stability of Alpha and Delta variants was greater at 4°C than at 24°C. The health impact is significant for both variants, with an increased likelihood of contacting a contaminated surface at lower temperatures than at high temperatures.

Viruses display different phenotypes under different environmental conditions, and SARS-CoV-2 is no exception to this. In solution, SARS-CoV-2 is highly stable at 4°C, showing only a 0·7 log_10_ reduction of infectious titer on day 14 (4). Chan et al. compared surface stability of SARS-CoV-2 HKU-001a obtained from a family cluster in January 2020, at 4°C and 20 °C to 25°C on glass. The virus survived for >14 days at 4°C compared with 3 to 5 days at 20°C to 25°C (14). Longer virus persistence observed by Chan et al. at both 4°C and 20°C compared with the data of the present study, may be a consequence of the difference in virus titers, surface materials used, or recovery media.

The study by Kratzel et al. is the closest report currently available for comparison with our study. Here, SARS-CoV-2/München-1.1/2020/929 was inoculated on unnamed metal discs and dried at 4°C, room temperature and 30°C. After 9 days, the median half-lives for each condition were 12.9 h, 9.1 h and 17.9 h, respectively. However, in contrast to our data, the authors concluded that SARS-CoV-2 does not display major differences at 4°C or room temperature (3). However, they acknowledge that their study design had not considered the effect of other factors, including relative humidity which works synergistically with temperature. The present study was conducted at a higher relative humidity (63% [24°C] and >85% [4°C]) compared with the study by Kratzel et al. which was done at 30% to 40% RH. With the present study, there was a significant increase in persistence of Alpha and Delta variants at lower temperatures compared with that of higher temperatures. A possible further study would be to investigate the effect of different RH ranges on the persistence of the virus at each temperature. The stability of SARS-CoV, a close relative of SARS-CoV-2, was found by Casanova et al. to be highest at 4°C and 20% RH compared with 50% and >80% RH at the same temperature on stainless steel (15).

Two limitations in the present study should be considered. The first is the use of high virus starting titers, as these may have a protective effect on enveloped viruses, reducing the rate of environmental decay (16). In addition, such titers may be higher than what is involved in actual human transmission, although actual quantities of SARS-CoV-2 in patient aerosols is yet to be estimated. The number of viral particles in influenza aerosols has been estimated to be around 10 to 100 viral particles, based on a measurements of viral RNA (17, 18). The use of higher starting titers in surface survivability investigations are key for the detection of small differences in recovered viability. Without them, trend analysis would be challenging.

The second limitation was having the 24°C coupons open to the flexible film isolator (FFI) airflow, as this might have caused an increased reduction in viability due to desiccation in comparison to the coupons within the fridge with static air.

In summary, the data from the present study shows that Alpha and Delta variants are more stable at a lower temperature compared with a higher temperature. Our results also show the possibility that the increased transmissibility of Delta variant compared to Alpha variant may be partly due to a higher persistence under the conditions studied.

## MATERIALS AND METHODS

### SARS-CoV-2 isolates.

Alpha variant (Human nCoV19 isolate/England/MIG457/2020) was prepared as described by Pottage et al. (8). Delta variant (HCM/V/078 P2 24MAY2021) at passage 2 was isolated by United Kingdom Health Security Agency (UKHSA) High Containment Microbiology Group (HCM) in Vero/hSLAMS with a titer of 7.8 × 10^5^. All handling of infectious SARS-CoV-2 was performed within a containment level 3 laboratory.

### Experimental procedure.

Coupon preparation, inoculation, exposure, and recovery was completed as documented previously (8). In summary, six stainless steel coupons per time point were inoculated with 20 μL droplets (2 × 10 μL) of each variant stock suspension within an FFI. These were immediately split into two groups; one group was left to dry under laboratory conditions and the second group was placed into a refrigerator at 4°C and left to dry (contained within the FFI). Sampling was conducted at each time point by placing coupons individually into 1 mL of complete minimum essential media (Gibco, USA) + 1% l-glutamine (Gibco, USA) + 1% non-essential amino acids (Gibco, USA) + 2% HEPES (Gibco, USA) + Fetal Calf Serum (Sigma-Aldrich, USA) with four glass beads (3 mm diameter), followed by vortex-mixing for 1 min before aliquoting and freezing at −80°C. Coupons were left in their respective environments for their exposure period. Time points of 0 h (immediately after inoculation), 2.5 h, 24 h, 48 h, 72 h, 96 h, 168 h, and 240 h were used in this study. The environmental temperature and relative humidity were recorded. For virus titration, samples stored at −80°C were first thawed to room temperature, then serially diluted in infection media (minimum essential media, Gibco, USA) + 1% l-glutamine (Gibco, USA) + 1% non-essential amino acids (Gibco, USA) + 3% HEPES (Gibco, USA) + antibiotic/antimycotic (Gibco, USA), and plaque-assayed on Vero-E6 cells, before staining and enumeration of plaques.

### Statistical analysis.

Censored normal linear regression of the logarithm of the titer was used on all time points up to and including the time that the minimum detectable limit was first attained. Investigation of persistence started with a three-way interaction between a quadratic function of time (on analysis scale), variant, and temperature. This was simplified, if the fit was not significantly worse as judged by the likelihood ratio test, to a linear function of time interacting with these factors, where statistical significance level was taken to be 5%. It was also compared with a cubic function of time interacting with these factors to ascertain if the quadratic trend was adequate. The procedure was repeated, omitting time point 0 and the Akaike information criterion was used to compare the fit of the models with and without this time point. The normality assumption was assessed using the Shapiro-Francia test and the quantile-quantile plot on the residuals of the noncensored data from these models. All statistical analysis and data manipulation were done in STATA, version 17.0.
